# A mixed methods analysis of cannabis use routines for chronic pain management

**DOI:** 10.1186/s42238-021-00116-7

**Published:** 2022-01-11

**Authors:** Kevin F. Boehnke, Laura Yakas, J. Ryan Scott, Melissa DeJonckheere, Evangelos Litinas, Suzanne Sisley, Daniel J. Clauw, David A. Williams, Jenna McAfee

**Affiliations:** 1 Anesthesiology Department, University of Michigan Medical School, Ann Arbor, MI USA; 2 Department of Family Medicine, University of Michigan Medical School, Ann Arbor, MI USA; 3 Independent Cannabis Consultant, Ann Arbor, MI USA; 4 Scottsdale Research Institute, Phoenix, AZ USA

**Keywords:** Medical cannabis, Mixed methods, Routes of administration, Dosing regimen, Chronic pain, Medication substitution

## Abstract

**Background:**

The wide heterogeneity of available cannabis products makes it difficult for physicians to appropriately guide patients. In the current study, our objective was to characterize naturalistic cannabis use routines and explore associations between routines and reported benefits from consuming cannabis.

**Methods:**

We performed a mixed methods analysis of *n*=1087 cross-sectional survey responses from adults with self-reported chronic pain using cannabis for symptom management in the USA and Canada. First, we qualitatively analyzed responses to an open-ended question that assessed typical cannabis use routines, including administration routes, cannabinoid content, and timing. We then sub-grouped responses into categories based on inhalation (smoking, vaporizing) vs. non-inhalation (e.g., edibles). Finally, we investigated subgroups perceptions of how cannabis affected pain, overall health, and use of medications (e.g., substituting for opioids, benzodiazepines). Substitutions were treated as a count of medication classes, while responses for both pain and health were analyzed continuously, with − 2 indicating health declining a lot or pain increasing a lot and 2 indicating that health improved a lot or pain decreased a lot.

**Results:**

Routines varied widely in terms of administration routes, cannabinoid content, and use timing. Overall, 18.8%, 36.2%, and 45% used non-inhalation, inhalation, and non-inhalation + inhalation routes, respectively. Those who used inhalation routes were younger (mean age 46.5 [inhalation] and 49.2 [non-inhalation + inhalation] vs. 56.3 [inhalation], *F*=36.1, *p*<0.001), while a higher proportion of those who used non-inhalation routes were female (72.5% non-inhalation vs. 48.3% inhalation and 65.3% non-inhalation + inhalation, *X*^2^=59.6, *p*<0.001). THC-rich products were typically used at night, while CBD-rich products were more often used during the day. While all participants reported similarly decreased pain, participants using non-inhalation + inhalation administration routes reported larger improvements in health than the non-inhalation (mean difference = 0.32, 95% CI: 0.07–0.37, *p*<0.001) and inhalation subgroups (mean difference = 0.22, 95% CI: 0.07–0.37, *p*=0.001). Similarly, the non-inhalation + inhalation group had significantly more medication substitutions than those using non-inhalation (mean difference = 0.62, 95% CI: 0.33–0.90, *p*<0.001) and inhalation administration routes (mean difference = 0.45, 95% CI: 0.22–0.69, *p*<0.001), respectively.

**Conclusions:**

Subgrouping medical cannabis patients based on administration route profile may provide useful categories for future studies examining the risks and benefits of medical cannabis.

**Supplementary Information:**

The online version contains supplementary material available at 10.1186/s42238-021-00116-7.

## Introduction

In the past two decades, thirty-six states have legalized medical cannabis, with chronic pain being the most common reason for obtaining a medical cannabis license (Boehnke et al. 2019a). As availability increases, so too has the variety of cannabis products, including cannabis flower, tinctures, concentrates, topical lotions/creams, and edibles (MacCallum and Russo 2018; Steigerwald et al. 2018)—products which are available in state-licensed medical cannabis dispensaries in the USA but are typically self-administered with little or no physician oversight (Boehnke et al. 2019b). These products have variable effect onset based on administration route, with inhalation of smoked or vaporized cannabis flower typically causing effects within 5–10 min while tinctures, edibles, and topicals can take anywhere from 15 min to 3 h to take full effect (MacCallum and Russo 2018). Products often contain one or more cannabinoids, most commonly Δ-9-tetrahydrocannabinol (THC) and cannabidiol (CBD). THC has analgesic and sleep-inducing effects (Johnson et al. 2010; Svendsen et al. 2004), but is responsible for cannabis-related harm and abuse potential (Volkow et al. 2014). In contrast, CBD is non-intoxicating (VanDolah et al. 2019), modulates THC’s psychoactivity (Freeman et al. 2019), and has anti-convulsant (Devinsky et al. 2017) and anxiolytic effects (MacCallum and Russo 2018; Crippa et al. 2011). Preclinical studies suggest that CBD has analgesic and anti-inflammatory activity (Philpott et al. 2017) and CBD is widely used for chronic pain (Boehnke et al. 2021a), including as a substitute for other medications (Boehnke et al. 2021b; Capano et al. 2019). However, clinical trials on CBD effects on chronic pain are mixed, with small trials showing improvements in temporomandibular joint disorder pain and neuropathic pain (Nitecka-Buchta et al. 2019; Dixon et al. 2019), but a large, recent clinical trial showing no improvements associated with CBD treatment among people with hand osteoarthritis or psoriatic arthritis (Vela et al. 2021). Cannabis potency has also increased, with average THC concentrations increasing from 3 to 17.1% and the ratio of THC:CBD increasing from 14:1 to >100:1 between 1995 and 2017 (Chandra et al. 2019), leading to concerns about THC-related harm.

This complex naturalistic context creates generalizability issues for cannabinoid clinical trials, in which dosing paradigms contrast sharply with naturalistic cannabinoid use (Fisher et al. 2020). Indeed, most clinical trials use products such as dronabinol (synthetic THC), nabiximols (a 1:1 THC:CBD sublingual spray not available in the USA), and cannabis grown at the University of Mississippi—none of which are representative of products available at medical cannabis dispensaries (National Academies of Sciences, Engineering, and Medicine 2017). These clinical trials also show small but significant effects on pain (Fisher et al. 2020) while consumers typically report much larger benefits and use for harm reduction (e.g., fewer side effects, better symptom management with cannabis) (Boehnke et al. 2019c). This mismatch, coupled with poor communication between physicians and patients as well as physician concerns about how to advise medical cannabis patients (Rubin 2017), demonstrates the importance of understanding naturalistic cannabis use. While medical cannabis patients with chronic pain use a variety of products and cannabinoid ratios (Boehnke et al. 2019b), the predominance of different use routines is uncertain and few studies have linked routines to clinical outcomes—a pressing question given the lack of rigorous clinical trial evidence supporting the use of CBD-rich products for chronic pain and reports of substituting cannabis for pain medications (Boehnke et al. 2019c; Lucas et al. 2019).

Thus, our primary objective was to better characterize and describe cannabis use routines for chronic pain. We employed a mixed methods design, which integrates qualitative and quantitative elements (Creswell and Clark 2006). First, we used a qualitative descriptive approach to describe typical cannabis use routines among individuals using cannabis for chronic pain management (Boehnke et al. 2019b; Boehnke et al. 2019c). We characterize routine heterogeneity by administration routes, cannabinoid content, use timing, and complexity of routine. After subgrouping cannabis use routines by administration routes, we explored relationships between subgroups and perceptions of cannabis effectiveness on pain, health, demographics, and medication substitution patterns—the latter in response to reports of patients substituting cannabis for pain medications (Boehnke et al. 2019c; Lucas et al. 2019).

## Methods

This is a secondary analysis of cross-sectional survey data conducted among adults (≥18 years old) in US states with legal medical/recreational cannabis or Canada who were self-administering cannabis for chronic pain symptoms. Cannabis dispensaries and certification clinics shared an anonymous Qualtrics (Provo, UT) survey link with their client databases and on social media between January and August 2018. Participants freely consented to participate, could drop out at any time, and were not compensated. We descriptively analyzed open-ended written responses to the following question, “Please describe your typical routine for using cannabis. For example, ‘I vaporize a high CBD cannabis distillate for pain relief throughout the day, and then take 10 drops of a strong THC tincture before bed.’” As with previous studies with this cohort (Boehnke et al. 2019b; Boehnke et al. 2019c), analyses initially included 1321 participants from a pool of 1697 responses (77.8% completion rate of the original survey). In the current study, participants were excluded if they either did not respond to the question (*n*=68, 5.1%), their responses were vague (e.g., “I take a maintenance dose”, *n*=48, 3.6%,) or they did not mention an administration route (*n*=118, 8.9%), resulting in 1087 responses (64.1% of the original *N*=1697 responses). Study procedures were approved as an exempt study (HUM00079274) by the Institutional Review Board at the University of Michigan due to the anonymous, confidential survey study design of this project.

### Qualitative descriptive analysis of routine data: Codebook creation

We created the codebook using an inductive descriptive approach whereby codes were developed based on respondent answers to identify variation in cannabis use routines (Sandelowski 2000). Descriptive analysis is a strategy to summarize participant experiences, focusing on their own words and how they relay their experiences. Codebook development involved two members of the research team (K.B., J.M.) reading through a sample of responses, independently developing a list of codes that described participant responses, and collaboratively developing a first draft of the codebook. The codebook included the list of codes, definitions of responses that fit each code, and methods for resolving discrepancies. Next, L.Y. coded the entire dataset, updating the codebook as necessary and meeting with the research team to discuss adjustments. After coding, we validated for inter-coder reliability. Ten percent of the entire data-set was selected for validation (Mao 2017) using the random number generator feature in Microsoft Excel, which K.B. then independently checked to confirm application of codes. After validation, we met to discuss discrepancies and resolve any responses that had created ambiguity. Our final codebook included multiple elements of use routines, including cannabinoid content, cannabis subtype, administration route, and timing. Brief descriptions of each component are below, and full descriptions may be found in the codebook (see Additional file 1: Appendix 1).

*Administration routes* included smoking, vaporizing, edibles, tinctures, topicals, and other. “Other” incorporated unspecified or rare administration routes and were not included in these categories.

*Timing* of use was coded as: Morning (before 12 PM); Afternoon (12–5 PM); Night (after 5 PM); and throughout the day (e.g., “every few hours,” “all the time”). We also created two non-specific codes: as needed and uncertain. As needed was used when language indicated that use is not routine but related to a specific symptom (e.g., “when I have spasms”). Uncertain was used when timing remained unclear (e.g., “I smoke”).

*Cannabinoid content* refers to CBD, THC, or the combination. When participants did not mention the type of cannabinoid, these responses were coded as “unknown cannabinoids.”

*Cannabis subtype* refers to indica, sativa, or hybrid cannabis.

### Subgroups

We created subgroups based on mutually exclusive categories of inhalation (smoking, vaping) and non-inhalation (edibles, tinctures, topicals). These groupings were chosen because effect onset is known to be quite different between administration routes (e.g., 5–10 min for smoking vs. 1–3 h for edibles), which may result in different effects and signify different use populations (MacCallum and Russo 2018). Indeed, inhalation routes are most commonly used and associated with more addiction/abuse potential and health risks but also more effective pain relief (MacCallum and Russo 2018; Andreae et al. 2015), while non-inhalation routes are used more by newer medical cannabis patients (Boehnke et al. 2019b).

### Measures for exploratory analysis

#### Demographic and clinical characteristics

We collected data on sex, age, socioeconomic status, concomitant self-reported current opioid and benzodiazepine use, alcohol use (never vs. ever drank), frequency of cannabis use (days/week, times/day), and cigarette use (never, former, and current use).

#### Changes in pain and health

We asked participants how their health and pain had changed since they started using medical cannabis, with response options on a five-point Likert scale from “Declined a lot” to “Improved a lot” for health and “Increased a lot” to “Decreased a lot” for pain. Responses for both pain and health were analyzed continuously, with − 2 indicating health declining a lot or pain increasing a lot and 2 indicating that health improved a lot or pain decreased a lot.

#### Medication substitution

As described previously (Boehnke et al. 2019c), participants reported classes of pain medication for which they substituted medical cannabis, including opioids, benzodiazepines, gabapentanoids, disease-modifying anti-rheumatic drugs, non-steroidal anti-inflammatory drugs, selective serotonin reuptake inhibitors, selective norepinephrine reuptake inhibitors, and other. The number of substituted medication classes was counted and analyzed continuously.

### Statistical analyses

Qualitative analyses are presented descriptively using representative quotes and counts of different cannabis use routine variables: e.g., administration routes, cannabinoid content, cannabis subtype, use timing. After subgrouping routines by administration routes (non-inhalation, inhalation, non-inhalation + inhalation), we explored differences for categorical variables (e.g., sex, income, tobacco, alcohol) using Pearson’s chi-square. For continuous variables (age, perceived changes in pain/health, substitutions), we assessed differences using one-way analysis of variance (ANOVA) followed by post hoc testing with Tukey’s test to identify subgroups driving significant differences of changes in pain, health, and substitution. Continuous variables were assessed for normality and were normally distributed, and are reported as mean +/− standard deviation. All tests were two-tailed, and significance set at *p* < 0.05. Analyses were performed using SPSS 25 (IBM, Armonk, New York) and Excel version 2019 (Microsoft, Redmond, Washington).

## Results

Participants (*N*=1087) were 60.5% female, 49.6±13.8 years old, and used medical cannabis for an average of 4.6±2.6 conditions or symptoms—most commonly severe and chronic pain (68.8%), back pain (58%) anxiety (51.9%), and depression (39.9%). Overall, *n*=405 (37.2%) had used cannabis for less than 1 year, while *n*=682 (62.8%) had used cannabis for more than 1 year. Routines were characterized as non-inhalation, inhalation, or non-inhalation + inhalation based on use of administration routes (Table 1).

**Table 1:** Examples of cannabis use routines by administration route

Subgroups	***n*** (%)	Example quote
Non-inhalation	204 (18.8%)	“2 drops of 1000mg CBD oil, 2x day.” “I take both THC & CBD tinctures in the AM, and usually a salve, then I put some Hash tincture in the water I have throughout the day. At night it can vary - sometimes just salve, sometimes nothing, sometimes an edible.”
Inhalation	393 (36.1%)	“I vape a sativa dominant hybrid in the morning and sometimes late afternoon. I smoke indica before bed.” “I smoke high THC out of a pipe every hour or two throughout the day.”
Non-inhalation + inhalation	490 (45.1%)	“I medicate with 5mg 2:1 CBD:THC gummy. I immediately vape 3 drags from a 2:1 cartridge. I then micro-dose the same at 2.5 mg every 2 hours with 2 hits off the 2:1 vape pen. When I get home after work, I take a 2mg THC blueberry and water-vape a very small mt of an indica. Then, before bed I water vape slightly more indica and take a 5mg dose of a THC/indica gummy.” “7 drops CBD twice daily, 2 drops 1:1 ratio before bed, vaporize higher THC as needed (esp. for neuropathic pain relief).”

*n* = 234 participants were excluded due to not responding, response vagueness, or because they did not mention administration routes. All groups are exclusive

*THC* tetrahydrocannabinol, *CBD* cannabidiol, *AM* morning

### Characterizing cannabis use routines

Cannabis use routines were quite variable in terms of complexity and what details were reported. For example, some participants reported very few details, “Smoke in morning, eat later on.” Others explicitly mentioned how they used different cannabinoids and products for specific symptoms at different times, “Sativa 1:1 cookie for daytime pain & Indica THC oil in tea at bedtime for sleep & pain.” Less than 6% of routines mentioned specific dosages in milligrams, and those who did often did so in the context of multiple administration routes, “Oral administration of high THC 14 mg plus High CBD/ THC combo 14 mg every morning. At bedtime 28 mg high THC/ CBD mix. PRN vape and bedtime application of lotion.” Although unprompted, 48 participants (4.4%) mentioned using cannabis products only in certain situations to avoid intoxicating effects: e.g., “any product w/THC ONLY if I am staying home” and “I do not use any cannabis products before or during work.” Some participants had a simple routine with 1–2 uses and/or administration routes: “I take a tincture about ½ hour before bed.” In contrast, others described complex routines involving multiple uses, administration routes, and cannabinoids at different times: “I use topical cannabis cream when I wake up and when I go to bed daily. Sometimes I require a mid-day application. I take 4 drops of CBD tincture morning and night for anxiety and sleep. I occasionally vape high CBD cannabis (3:1 or greater) for breakthrough pain.” Examples of the variety of routine complexity are displayed in Table 2.

**Table 2:** Examples of routines with different complexity

	Subgroup	Administration routes	Timing
**Simple routine**			
I take a tincture about 1/2 hour before bed.	Non-inhalation	Tincture	PM
I usually take 1 hit from the vape pen after work and another when I’m ready to sleep.	Inhalation	Vaporize	PM
I smoke for immediate relief and use tincture at bedtime for sleep	Non-inhalation + inhalation	Smoke, tincture	AM, PM
I smoke high CBD cannabis, indica/sativa blend for pain relief before dinner on the weekends. (Weeknights on as needed basis)	Inhalation	Smoke	PM, as needed
8 drops of CBD tincture before bed	Non-inhalation	Tincture	PM
**Moderate routine**			
I vaporize a high THC and CBD cannabis distillate for pain relief and then use a THC relax patch and vaporize in the evening	Non-inhalation + inhalation	Vaporize, topical	As needed, PM
I smoke high CBD in the morning upon getting up and ready. In the evening, I smoke a higher THC content Indica with CBD infusion prior to sleep.	Inhalation	Smoke	AM, PM
I vaporize 1 puff of high THC, low CBD cannabis after work for pain relief, and ingest a 1/4 grain of high THC indica in RSO form 1 hour before bedtime, which helps with insomnia due to PTSD.	Non-inhalation + inhalation	Vaporize, edible, concentrate	PM
1 edible with breakfast and 1 edible before dinner.	Non-inhalation	Edible	AM, PM
I use almost exclusively at night (after 10 PM) for pain and to help with sleep. I eat approx. 5-7 mg THC of edible first while watching TV and follow 1-2 hours later with 2-3 hits from handheld vaporizer before going to bed.	Non-inhalation + inhalation	Edible, vaporize	PM
**Complex routine**			
Use CBD tincture and vape during the day. If I get a migraine, I will use THC provided that I do not drive. At night I use a very high dose of indica THC tincture to try and stay asleep, and vaporize the same THC indica to try and fall asleep	Non-inhalation + inhalation	Tincture, Vaporize	Throughout the day, PM, as needed
In the morning, I ingest 70mg of CBD, apply tincture to my face and forearms and apply transdermal balm that I have strengthened with additional CBD oil to my feet and other areas. During the day I may vape CBD and apply additional CBD balm to my lips, arms, etc. Evening/night I continue the transdermal applications to my lips & skin. Before bed I vape a heavy Indica 2-4 inhalations and apply CBD oil to my hair part and face.	Non-inhalation + inhalation	Vaporize, Edible, Topical	AM, throughout the day, PM
I take a pure CBD tincture every morning (5-10 mg) and sometimes throughout the day for breakthrough pain. During the day I usually avoid THC if I am working, but will take high CBD, low THC tinctures when needed. In the evening I often take a 1:1 tincture or will take CBD oil/tincture and may have an edible or vaporize THC with friends. I also often use a 1:1 tincture before exercise or take CBD oil with a sativa strain vaporized (occasionally smoked from a glass bowl if I am out hiking or something).	Non-inhalation + inhalation	Tincture, vaporize, edible, smoke	AM, throughout the day, PM, as needed
I vaporize high CBD and sativa strains in the morning and throughout the day to control the onset and duration of effect. I use high THC in the evening via RSO pills with some vaporization or smoking around mealtimes. CBD tincture at bedtime seems to produce less mental fog in the morning.	Non-inhalation + inhalation	Vaporize, smoking, edible, tincture, concentrate	AM, throughout the day, PM
Morning: High CBD tincture (8:1 or 12:1), administer about 10mg CBD; Afternoon: As needed, usually a small dose 3:1 to 1:1 CBD:THC via tincture or vaporizer; Evening: High THC flowers or concentrate, smoke or vaporize, as needed for dinner digestion and sleep.	Non-inhalation + inhalation	Tincture, vaporize, smoke	AM, afternoon, PM
I use topical cannabis cream when I wake up and when I go to bed daily. Sometimes I require a mid-day application. I take 4 drops of CBD tincture morning and night for anxiety and sleep. I occasionally vape high CBD cannabis (3:1 or greater) for breakthrough pain.	Non-inhalation + inhalation	Topical, tincture, vaporize	AM, afternoon, PM, as needed

Routines varied in complexity, with some participants using numerous administration routes and cannabinoids at different times of the day

*THC* tetrahydrocannabinol, *CBD* cannabidiol, *AM* morning, *PM* evening, *RSO* Rick Simpson Oil, *PTSD* Post-traumatic stress disorder

Table 3 descriptively presents the distribution of use timing for administration routes, cannabinoids, and cannabis subtypes. Inhalation methods were more commonly used than non-inhalation methods and were most frequently used at night (36.7%) and throughout the day (24.9%). In contrast, non-inhalation administration routes were most often used at night (34.7%) and in the morning (19.4%). CBD was most often used in the morning (27.3%), throughout the day (25.2%), and evening (21.3%), while THC was most often used in the evening (38.9%) and throughout the day (15.3%). Cannabis subtype uses were mentioned with far lower frequency than administration routes and specific cannabinoids. Sativas were used more in the morning (32.2%) and throughout the day (37.1%), while Indicas were predominantly used at night (80.8%). Hybrids were used more throughout the day (30.6%) and evening (30.6%).

**Table 3:** Timing of use with regards to administration route categories, cannabis sub-types, and cannabinoids

	AM	PM	Afternoon	As needed	Throughout the day	Uncertain	Total
**Subgroups**							
**Inhalation**	146 (10.8%)	496 (36.7%)	71 (5.3%)	211 (15.6%)	337 (24.9%)	91 (6.7%)	1352
**Non-inhalation**	210 (19.4%)	377 (34.7%)	63 (5.8%)	168 (15.5%)	160 (14.7%)	107 (9.9%)	1085
**Cannabis subtypes**							
**Indica**	6 (2.7%)	177 (80.8%)	5 (2.3%)	13 (5.9%)	11 (5.0%)	7 (3.2%)	219
**Sativa**	46 (32.2%)	10 (7.0%)	14 (9.8%)	14 (9.8%)	53 (37.1%)	6 (4.2%)	143
**Hybrid**	4 (6.5%)	19 (30.6%)	11 (17.7%)	5 (8.1%)	19 (30.6%)	4 (6.5%)	62
**Cannabinoids**							
**CBD**	128 (27.3%)	100 (21.3%)	31 (6.6%)	56 (11.9%)	118 (25.2%)	36 (7.7%)	469
**THC**	44 (12.0%)	179 (48.9%)	15 (4.1%)	49 (13.4%)	59 (16.1%)	20 (5.5%)	366
**1:1 CBD:THC**	15 (21.1%)	20 (28.2%)	8 (11.3%)	8 (11.3%)	18 (25.4%)	2 (2.8%)	71
**Unknown cannabinoids**	184 (13.3%)	504 (36.3%)	82 (5.9%)	219 (15.8%)	278 (20.0%)	121 (8.7%)	1388

Timing of administration route categories, chemovars, and cannabinoids. Smoking and vaping were categorized as “inhalation,” while edibles, tinctures, and topicals were categorized as “non-inhalation.” “Other” and “Concentrates” were not subgrouped into inhalation or non-inhalation categories.

*AM* morning, *PM* evening

### Demographics and substances use patterns

Demographic characteristics and use of benzodiazepines, opioids, cigarettes, and alcohol among subgroups are described in Table 4. The distribution of administration routes varied significantly by sex and income, with more women in non-inhalation subgroups and more participants in the inhalation subgroups in a lower-income category. Average age varied significantly: those in the non-inhalation subgroup were older than participants in the inhalation (mean difference = 9.8, 95% CI [7.1–12.5], *p* < 0.001) and non-inhalation + inhalation subgroups (mean difference = 7.2, 95% CI [4.6–9.8], *p* < 0.001). The distribution of cigarette use also varied significantly, with more former or current cigarette smoking among inhalation subgroups than non-inhalation administration subgroups. Participants using non-inhalation administration routes alone used cannabis less frequently each day than those using inhalation or non-inhalation + inhalation routes. No statistically significant differences were seen in the distribution of alcohol use and current prescription benzodiazepine or opioid use.

**Table 4:** Demographics and substance use patterns of cannabis use routine subgroups

	Total (***n***=1087)	Non-inhalation (***n***=204)	Inhalation (***n***=393)	Non-inhalation + inhalation (***n***=490)	***X***^**2**^	***F***	***p***
**Sex**							
Female	60.5%	72.5%	48.3%	65.3%	59.6		<0.001
**Age** Mean (SD)	49.6 (13.8)	56.3 (11.7)	46.5 (14.0)	49.2 (13.5)		36.1	<0.001
**Annual income ($US)**							
<$10,000	6.9%	6.1%	9.1%	5.4%	67.7		0.002
$10,000–$39,999	29.8%	28.4%	35.2%	26.0%			
$40,000–$69,999	24.3%	24.4%	21.6%	26.4%			
$70,000–$99,999	18.9%	15.2%	16.9%	21.9%			
$100,000–$149,999	11.6%	13.2%	11.7%	11.0%			
More than $150,000	8.5%	12.7%	5.5%	9.3%			
**Cigarette use**							
Never	34.7%	48.6%	31.7%	31.7%	38.4		<0.001
Former	47.8%	41.7%	46.2%	51.3%			
Current	17.6%	9.7%	22.1%	17.0%			
**Alcohol use**							
Never drinker	27.9%	28.2%	28.3%	27.5%	7.6		0.48
Drinker	72.1%	71.8%	71.7%	72.5%			
**Opioids**							
Yes	15.8%	16.7%	13.6%	17.1%	9.9		0.26
**Benzodiazepines**							
Yes	12.7%	11.1%	11.8%	14.0%	5.2		0.72
**Cannabis use frequency**							
Days/week (mean/SD)	6.4 (1.4)	5.9 (1.8)	6.5 (1.3)	6.4 (1.2)		9.1	<0.001
Times/day (mean/SD)	3.1 (1.4)	2.2 (1.2)	3.3 (1.4)	3.3 (1.3)		13.0	<0.001

Demographics of each cannabis use routine subgroup

*SD* standard deviation

### Clinical outcomes: exploratory analyses

Most individuals reported decreased pain since initiation of cannabis use (Table 5), with no significant differences between groups *(p*=0.066). In contrast, there were significant differences in both reported health changes and number of substitutions between subgroups (both *p*’s < 0.001). These effects were driven by the non-inhalation + inhalation subgroup, which reported larger improvements in health than the non-inhalation (mean difference = 0.32, 95% CI: 0.07–0.37, *p* < 0.001) and inhalation subgroups (mean difference = 0.22, 95% CI: 0.07–0.37, *p* = 0.001). Those in the non-inhalation + inhalation subgroup also reported more medication substitutions on average than the non-inhalation (mean difference = 0.62, 95% CI: 0.33–0.90, *p* < 0.001) and inhalation subgroups (mean difference = 0.45, 95% CI: 0.22–0.69, *p* < 0.001).

**Table 5:** Self-reported changes in pain and health since initiation of cannabis use

	Total (***n***=1087)	Non-inhalation (***n***=204)	Inhalation (***n***=393)	Non-inhalation + inhalation (***n***=490)	***F***	***p***
**Pain** Mean (SD)	− 1.45 (0.78) *n*=1052	− 1.41 (0.80) *n*=191	− 1.4 (0.81) *n*=382	− 1.51 (0.75) *n*=479	2.7	0.066
**Health** Mean (SD)	1.05 (0.92) *n*=1050	0.87 (0.92) *n*=190	0.97 (1.19) *n*=382	1.19 (0.89) *n*=478	10.8	<0.001
**# Subs** Mean (SD)	1.72 (1.45) *n*=1049	1.40 (1.21) *n*=191	1.56 (1.53) *n*=380	2.01 (1.46) *n*=478	17.1	<0.001

Self-reported changes in pain and health among subgroups of cannabis use routines

## Discussion

Our investigation provides a novel view into cannabis use routines for chronic pain management. Our mixed methods design showcases the immense breadth of cannabis use routines, including administration routes, cannabinoid content, and timing. Our exploratory analyses provide enticing hints of how routines may affect health and medication substitution outcomes, as use of non-inhalation + inhalation administration routes was associated with greater self-reported improvements in health and more substitutions for pain medications.

### Common trends in cannabis use routines

First, we show the great heterogeneity of cannabis use routines for chronic pain, with some participants using a single administration route and the same type of cannabinoid once per day, while others used multiple administration routes, cannabinoids (CBD, THC, or unknown), and subtypes variably throughout the day. This heterogeneity likely reflects both the large variety of cannabis products available as well as the wide array of factors (e.g., past recreational use, age) that influence how people decide to use cannabis.

Second, 45.0% of respondents used non-inhalation + inhalation administration routes, compared to 36.2% using inhalation administration routes alone and 18.8% using non-inhalation administration routes alone. As tinctures/edibles take effect more slowly than inhalation (MacCallum and Russo 2018), this finding suggests that many participants may be layering administration routes to achieve tailored symptom relief. It is also in alignment with recent findings that adults >50 years (the majority of our study population) are more likely to use non-inhalation administration routes (Kaufmann et al. 2020). Similarly, the demographics of those in the inhalation subgroup (higher % male, younger, lower-income, more former/current cigarette smoking than non-inhalation subgroups) is consistent with cannabis use patterns countrywide (Compton et al. 2016). The increased daily frequency of use among subgroups using inhalation administration routes is also congruent with known pharmacokinetic effects, as smoking and vaporizing do not produce as long-lasting results as edibles or tinctures (MacCallum and Russo 2018).

Third, use timing was not uniform: while ~35% of uses occurred at night, use at other times of day varied depending on administration route, cannabinoid content, and most strikingly, cannabis subtype. The overwhelming use of Indicas at night matches the popular belief that Indicas are sedating, despite the fact that these categories lack scientific validity (Piomelli and Russo 2016; Watts et al. 2021). This latter point emphasizes the need for synchronizing the scientific literature with the (largely unregulated) cannabis market in which this misconception is playing out.

Fourth, most participants did not mention which cannabinoids they were taking, possibly due to a lack of knowledge around content as there is inconsistent labeling among medical cannabis products. It is also possible that people did not mention cannabinoids as cannabis effects are typically associated with THC intoxication and THC-containing products are the most common in the medical cannabis market (Gurley et al. 2020). Taken together, these trends highlight the importance of carefully distinguishing use patterns among medical cannabis patients and also educating patients around differences between cannabinoid effects.

### Self-reported changes in pain, health, and medication substitution

While subgroups showed no significant differences in perceptions of how their pain had changed, the non-inhalation + inhalation subgroup reported significantly higher improvements in health and more substitutions of cannabis for pain medications compared to those using just non-inhalation or inhalation subgroups. This difference could be due to negative health effects of solely smoking/vaporizing cannabis (Volkow et al. 2014) or difficulty titrating non-inhalation administration routes (especially high-potency edibles (Steigerwald et al. 2018)), which can result in adverse reactions, as demonstrated by increased rates of emergency room visits related to edible cannabis products (Monte et al. 2019). This latter point is also important as the average age of the non-inhalation group was nearly a decade higher than that of the other subgroups, potentially reflecting factors (e.g,. polypharmacy, possible drug-drug interactions, more co-morbidities) that uniquely affect this older population (Mahvan et al. 2017). In contrast, using non-inhalation administration routes (e.g., edibles) analogously to extended release medications for long-term relief and inhalation administration routes for breakthrough symptoms may result in more targeted pain relief if done judiciously (Boehnke et al. 2019b). This potentially indicates a better understanding of cannabis products among these participants or (as shown in other reports) use for harm reduction (Boehnke et al. 2019c; Lucas et al. 2019; Lau et al. 2015). Indeed, although not addressed in our prompt, 48 participants indicated that they use certain products only in specific situations to minimize harm—e.g., not when working, or only at home. This harm-reduction tendency is also reflected in timing trends, as the most common timing of use for any administration route was at night. In addition, CBD (non-intoxicating (VanDolah et al. 2019)) was used more in the morning and during the day while THC (intoxicating) was used more in the evening.

### Inference for broader clinical and cannabinoid literature

The heterogeneity of use routines may partially explain variable findings among observational studies of individuals using medical cannabis. A prime example is the debate around whether cannabis could be an effective substitute for pain medications. While individuals in some studies report improved pain and substituting cannabis for pain medications (Lucas et al. 2020; Aviram et al. 2020), others show greater pain severity and risk of pain medication use and misuse (Caputi and Humphreys 2018; Campbell et al. 2018). However, none of these studies assessed how use patterns might contribute to differential substitution success. It is plausible that the disparate effects were driven by subsets of individuals, rendering both claims true. Indeed, the exploratory findings from our study suggest that individuals using a mixture of administration routes reported improved health and substituted cannabis for more pain medications than those in most other subgroups. Our results highlight the importance of performing a thorough medical cannabis assessment, as the details—rather than a simple binary yes/no—are more likely to reveal harmful vs. beneficial use. Similarly, our findings point towards the need for high-quality, prospective observational studies to complement the clinical trials literature, which has numerous acknowledged shortcomings including short interventions, minimal studies with CBD alone, and dosing regimens that are not representative of naturalistic use (Fisher et al. 2020).

### Limitations

First, recruitment at dispensaries may have resulted in selection bias, influencing use routines and perceptions of cannabis effectiveness. Second, our cross-sectional design renders perceived effects of cannabis on pain and health subject to recall bias, especially among participants who had been using cannabis for years. Pain and health were also not assessed using a psychometrically validated measure. However, Likert scales are commonly used in the pain literature to assess pain symptoms (Dworkin et al. 2008). Third, we asked more about the cannabis use routine rather than the rationale for use, which may limit the quantity of detail that participants provided. In addition, our question did not specify the time frame of routine: e.g., daily vs. weekly. Fourth, our inability to categorize participants based on cannabinoids likely underestimates the true use of THC, but the overwhelming dominance of the “unknown cannabinoid” category renders this categorization impractical with our data. Fifth, we could not estimate dose nor did we have any laboratory testing to provide additional information on what products people used, which may have affected our results. However, given the heterogeneity of cannabis products and uncertainty around the specific content of these products caused by inaccurate labeling (Vandrey et al. 2015; Bonn-Miller et al. 2017), we are uncertain that dose estimates would necessarily provide us with accurate information. Sixth, our results have uncertain external validity as we did not ask participants where they had heard about the survey (e.g., dispensary vs. clinical care) and were thus unable to characterize differences based on those who received care/information in a clinic vs. in a dispensary. Similarly, we are uncertain which dispensaries and the total number of participants who had access to the survey. Seventh, due to our anonymous, confidential survey design, participants self-reported their use of cannabis for chronic pain, but we were unable to verify whether they were diagnosed by a physician.

## Conclusions

Our mixed methods approach revealed immense heterogeneity of cannabis use routines for chronic pain. Subgrouping medical cannabis patients based on administration routes and cannabinoid use profile may provide useful categories for examining cannabinoid effects in future research. Future longitudinal studies should characterize how use trends affect pain management and safety outcomes among people with chronic pain of various etiologies.

## Supplementary Information

**Additional file 1: Appendix 1**. Codebook for qualitative descriptive analyses.

**Additional file 2:.** STROBE checklist.

## Data Availability

Data available from authors by reasonable request.

## Abbreviations

AM: Morning
CBD: Cannabidiol
CI: Confidence interval
PM: Evening
PRN: Pro re nata
THC: Δ-9-tetrahydrocannabinol
