# Supplementary material for: A mixed methods analysis of cannabis use routines for chronic pain management
Source: J Cannabis Res. 2022 Jan 11;4:7. doi: 10.1186/s42238-021-00116-7 (PMC8750808; doi:10.1186/s42238-021-00116-7)
Supplement: Supplementary file 1 — Additional file 1: Appendix 1. Codebook for qualitative descriptive analyses. [file 42238_2021_116_MOESM1_ESM.docx]

**Codebook for qualitative descriptive analyses:**

**Administration Routes:** Administration route refers to the way in which cannabis was ingested.

**Smoking:** smoke, joint, bong, blunt, water pipe, combust, toke, hit

**Vaporizing**: vape pen, vaporizing, vape, volcano, cartridge

**Edibles:** cookie, brownie, gummy, “ingest”, edible, “eat”, “drink”, capsule, candy, lozenge, macaroons, juice, MCT, chew, oil, syrup

**Concentrates:** concentrate, distillate, RSO, Rick Simpson Oil, hash, shatter, extraction, dab, syringe, feco or F.E.C.O. or full extract cannabis oil, liquid blend. Concentrates were coded as well as any other administration route mentioned when relevant. I.e., “I smoke hash” would be coded for both “smoke” and “concentrate”. If mentioned without an administration route, then coded as “other” administration.

**Tincture:** drops, tincture, sublingual, under the tongue, solution, Trokie (a lozenge that dissolves under lip/is similarly fast acting)

**Topical:** lotion, salve, cream, oil (if language states applied topically), spray, patch, rub, roll-on, balm, transdermal, body application, apply

**Other administration:** used for rare administration routes (e.g., suppository) or when administration route is not mentioned (i.e., I take CBD)

**Oil:** if language indicates smoked, topical, vaporized, eaten, or tincture, put it in those categories. Otherwise, counted as “other administration’

**Timing:** *Timing* refers to approximate time of day of cannabis use. Specific timing codes included: Morning, Afternoon, PM, and throughout the day. Use of the same administration route and product in the morning, afternoon, and evening was coded as throughout the day. However, if a person used the same administration route but different products (e.g., smoked sativa in morning/afternoon and indica in the evening) each time point would be coded (i.e., Smoking AM, afternoon, and PM). In addition, we created two non-specific codes: PRN (“as needed”), and Uncertain. PRN was used when no specific time was mentioned and language indicated that use is not routine but related to a specific symptom (e.g. “occasionally” or “sometimes” using cannabis for “pain” or “sleep”). If a time was mentioned, that time was foregrounded, even if “as needed” or PRN was written. We also used PRN to account for routines that changed depending on weekly schedules, e.g., participants who only used cannabis on weekends. We coded Uncertain Timing when responses did not mention timing (e.g. “I smoke”) or timing remained uncertain (e.g., “when I have it”, “when I am at home”, or “once/twice a day”).

**AM:** morning, before noon, any time mentioned with AM, after waking, when I wake up, after getting out of bed, 5am-<12pm, early, early in the day

**Afternoon:** noon, afternoon, before dinner, 12pm-5pm

**Evening:** After 5pm, after dinner, before bed, after school/after work, night, bedtime, evening, late, late in the day

**Throughout the day**: throughout the day, “3-4 times per day”, “every 4-6 hours”, during the day, each day, morning noon and night, all the time.

**PRN:** as needed, if needed, when I have (symptom), If I feel awful, if I feel (symptom) and need it, occasionally/periodically/sometimes (if it makes sense by context i.e., related to symptom, otherwise uncertain), if necessary, break-through (symptom), PRN, also for any mention of routines that differ “on the weekend” (i.e. I smoke during the day on weekends only would be smoke PRN), x times per week (e.g., 3-4 times per week), mixed in, supplemented with

**Uncertain (timing):** sometimes (unless PRN, or if they list a time – i.e. “sometimes at night” – or if it seems by context that the “sometimes” is an “or”- i.e. “I smoke a night, sometimes take an edible too”), when I can afford/budget allows/I can find it, from time to time, if nothing is mentioned or absence of other information besides administration route (i.e., if someone says, “I smoke”), occasionally (when signified as alternative routine, otherwise PRN) once per day or twice per day, “when at home”, typically (unless associated with a time)

NOTE: If someone says, “I eat a cookie (or other long-lasting product) in the morning to last through the day”, we chose morning rather than throughout the day.

**Cannabinoids:** We developed specific rules to account for ambiguous wording: e.g., a response of “high CBD” without mentioning THC was coded as “High CBD: Low THC,” as we determined the words “high” and “low” to be a signals of cannabinoid ratio. When a participant mentioned a “blend,” “mix,” or “both” CBD and THC without a ratio indication, we coded both “THC” and “CBD”.

**CBD:** cannabidiol, CBD, ACDC (a CBD-dominant strain), or CBD dominant

**THC:** THC, THCA, marinol, dronabinol, tetrahydrocannabinol, “Potent/strong”

**Ratio:** If participant mentions “high” or “low” in relation to CBD or THC, use the category of ratio. That is, if a participant mentions high CBD but no mention of THC, assume high CBD: low THC. However, if a participant mentions a mix or blend of CBD and THC or “CBD and THC,” or CBD/THC, without specifying the ratio, code as both CBD and THC categories. 50/50=1:1 CBD:THC. 0.8:1 or closer is specified as 1:1.

**Unknown:** If no mention of CBD or THC, just an administration route (“I smoke” or “edible”), response was placed into “unknown cannabinoid” category

**Cannabis Subtype**: Refers to indica, sativa, or hybrid subtypes of cannabis. Indica or sativa categories included qualifiers such as “a blend with higher sativa”, whereas we would select hybrid for a response that mentioned both indica and sativa without a ratio indication, similar to the above THC/CBD rule.

**Indica:** includes indica dominant, or “indica hybrid” or “indica blend”

**Sativa:** includes sativa dominant, “blend with higher sativa” “sativa blend”

**Hybrid:** includes “sativa indica blend” and also just “blend” in context

**Miscellaneous**

If participant stated, “I used to do _______”, then this is not counted. This includes “Prior to this time” if the person lists a current routine. If participants mentions a future hypothetical like “I’m thinking about” or “I’m going to try” then discounted. The context of “would like to” or “would prefer to” also indicates that they do not have a specific routine because they WOULD do it instead of are actually doing it.

If grammar was uncertain, we coded the response but flagged it as difficult to interpret so it could be resolved by two or more investigators at a later date.

Qualifiers like “more” and “mild” did not affect coding because those appear to be dosing related rather than cannabinoid content or ratio related.

**No routine:**

Participants were categorized as not having a routine if what they described was not considered a routine. E.g., “It varies. I never do the same thing twice. It depends on what’s available.”
